# Evaluation of an autologous cancer vaccine for the treatment of metastatic canine hemangiosarcoma: a preliminary study

**DOI:** 10.1186/s12917-020-02675-y

**Published:** 2020-11-18

**Authors:** Michael D. Lucroy, Ryan M. Clauson, Mark A. Suckow, Ferris El-Tayyeb, Ashley Kalinauskas

**Affiliations:** 1 Torigen Pharmaceuticals, Inc, 400 Farmington Avenue R1855 CB129, 06032 Farmington, CT USA; 2 Office of the Vice President for Research, University of Kentucky, 445 Bowman Hall, KY 40506-0032 Lexington, USA

**Keywords:** Immunotherapy, Canine hemangiosarcoma, CD80, MCHII, Autologous cancer vaccine

## Abstract

**Background:**

Canine hemangiosarcoma (HSA) is an aggressive cancer arising from multipotential bone marrow-derived stem cells. Anthracycline chemotherapy drugs have been the mainstay adjuvant chemotherapy following surgery with only modest improvement in survival and an attendant risk for adverse events. Immunotherapy, using a whole cell autologous cancer vaccine adjuvanted with MIM-SIS, may improve outcomes for dogs with HSA with a lower risk for adverse events compared with chemotherapy.

**Results:**

In cultured DH82 canine monocyte-like cells, autologous cancer vaccines prepared from 13 dogs with HSA increased MHC-II surface expression ranging from 20.0-60.4% on single-stained cells, CD80 surface expression ranging from 23.7–45.9% on single-stained cells, and MHC-II/CD80 surface expression ranging from 7.2–20.1% on double-stained cells. Autologous cancer vaccines were able to, on average, stimulate an up-regulation of MHC-II and CD80 by 48-fold as compared to media only (MHC-II + CD80 + cells: 12.19 ± 3.70% vs. 0.25 ± 0.06%; *p* < 0.001). The overall median survival time for dogs treated with the autologous cancer vaccine was 142 days (range, 61 to 373 days). Dogs treated with the autologous cancer vaccine or maximum tolerated dose (MTD) chemotherapy had significantly (*P < 0.001)* longer survival than dogs treated with surgery alone. The 1-year survival rate was 12.5% for dogs treated with the autologous cancer vaccine, and 0% for dogs treated with surgery alone or MTD chemotherapy. No adverse events were observed in the dogs treated with the autologous cancer vaccine.

**Conclusions:**

The adjuvanted autologous cancer vaccine is capable of up-regulating MHC-II and CD80 in cultured canine monocyte-derived cells, which are important stimulatory molecules in generating an immune response and improves survival time in dogs with metastatic (stage III) HSA when compared to surgical treatment alone. Autologous cancer vaccine-treated dogs had survival similar to those dogs treated with MTD chemotherapy without any observed adverse events. This autologous cancer vaccine represents an effective form of individualized immunotherapy that is an appealing option for dog owners not wanting to pursue adjuvant chemotherapy for HSA.

## Background

Canine hemangiosarcoma (HSA) is an aggressive, ultimately fatal, cancer arising from multipotential bone marrow-derived stem cells that arrest their differentiation at the hemangioblast or angioblast stage [1]. Based on gene expression profiling revealing upregulation of VEGF, MMPs, TIMPs, etc. and enrichment of cytokines, it appears inflammation and angiogenesis are two important processes in the pathogenesis of canine HSA [2]. The HSA microenvironment has also been shown to enhance tumor growth and promote migration of tumor cells through chemokines such as IL-8 and CXCL12 and modified sphingosines [3]. The spleen is the most common site of HSA in the dog, although liver, heart, and skeletal muscle may also be sites of origin [3, 4]. After splenectomy, or wide surgical excision at other sites, reported median survival times range from 19 to 110 days, with death typically due to widespread metastasis which often results in significant hemorrhage [4–10]. Dogs with metastatic disease at the time of diagnosis (stage III) have a poor prognosis, with reported median survival times from 23 to 40 days following surgical removal of the primary tumor [5, 9, 10].

For nearly 30 years, anthracycline chemotherapy drugs (most notably doxorubicin) have been the mainstay adjuvant chemotherapy following splenectomy, and modestly prolongs survival of affected dogs. When combined with surgery, doxorubicin-based chemotherapy protocols have reported median survival times ranging from 140 to 202 days [11]. Epirubicin, another anthracycline drug, has a reported median survival time of 144 days when used to treat canine splenic HSA after splenectomy [12]. Combining doxorubicin with other chemotherapy drugs does not typically prolong patient survival, and additions of toceranib, vincristine with cyclophosphamide, or metronomic cyclophosphamide, have reported median survival times of 172, 145 and 202 days, respectively, in populations of dogs that included those without metastatic disease (stage I and II) [13–15]. However, the addition of dacarbazine has been reported to increase the median survival time to > 550 days in a group of 9 dogs with stage II HSA [16]. Doxorubicin chemotherapy is modestly effective for dogs with stage III HSA, extending the median survival time to 107 to 140 days [10, 17]. Altered expression of the ATP-binding cassette transporters ABCB1 and ABCG2 may be partly responsible for the inherent drug resistance observed in canine HSA [18].

In addition to the potential for prolonging survival in dogs with HSA, chemotherapy also has the potential to cause adverse events. In dogs with stage III HSA treated with anthracycline chemotherapy, 43.5% had adverse events, with 21.7% of treated dogs requiring hospitalization for supportive care [10]. The combination of doxorubicin, vincristine and cyclophosphamide (VAC protocol) to treat canine HSA caused neutropenia in 73% of dogs, 27% had severe gastrointestinal (GI) adverse events, and 13% developed sepsis [14]. Epirubicin is also reported to have a high rate of adverse events when used to treat cancer-bearing dogs [19]. Despite the positive effect on survival for cancer-bearing dogs, recent findings show that the majority of surveyed pet owners would not pursue chemotherapy due to concerns about the risk for adverse events [20].

Immunotherapy is another treatment option that may improve patient outcomes for dogs with HSA with a lower risk for adverse events compared with chemotherapy. An allogenic vaccine, consisting of lysates from two canine HSA cell lines and a liposome-DNA adjuvant, was administered to 28 dogs with various forms of HSA via intraperitoneal (IP) injection [21]. In subset analysis of 13 dogs with stage II HSA treated with only doxorubicin and the vaccine, the reported median survival time was 182 days, which is similar to chemotherapy alone. A dendritic cell vaccine was evaluated in 5 dogs with splenic HSA [22]. Monocytes isolated from peripheral blood were incubated with tumor-derived mRNA to create the vaccine, which was administered via IP injection. The dogs were also treated with doxorubicin (20 mg/m^2^). The reported median survival time was 109 days. Both of these vaccine approaches were reportedly well-tolerated.

Autologous tissue vaccine is another immunotherapy option for dogs with HSA. Autologous tissue vaccines consist of cells harvested from the patient’s own tumor, which presents the full repertoire of the patient’s unique tumor-associated antigens (TAA) to the immune system. A method of creating autologous tumor vaccines for dogs has been recently described, and relies on enzymatic cell dissociation, which could destroy TAA [23]. Although some humoral response was described in the treated dogs, survival outcome was not reported for the single dog with HSA in that study population.

Another method of autologous cancer immunotherapy is a whole cell autologous cancer vaccine, described herein. Cancer cells reproduce within a mesh of supporting tumor stroma, and reciprocal interactions between neoplastic cells and their surrounding microenvironment are critical factors in tumor progression. Many anticancer immunotherapies have focused on persuading the host immune system to recognize specific TAA; however, the ability for tumor destruction by such vaccines has been disappointing, perhaps because TAA are lost over time in the growing tumor, do not represent the full repertoire of antigens, and/or the host grows tolerant to them. For this reason, we chose an approach that presents the immune system with a broad menu of antigen targets, including those that are associated with neoplastic cells and those that are associated with the tumor stroma that is essential to tumor growth and spread.

This whole cell autologous cancer vaccine is generated by a novel method of mechanically dissociating tumor cells followed by chemical inactivation, to preserve TAA, and then mixing the cellular material with MIM-SIS (matrix immunomodulator, small intestine submucosa-derived), a protein immune adjuvant [24]. Extracellular matrix bioscaffolds used in tissue and wound repair applications are pro-inflammatory, and modulation of macrophage phenotype is believed to play an important role in the mechanism of action [25]. Because of this ability to drive the immune response, medical grade SIS has been evaluated for use as an adjuvant for therapeutic cancer vaccines. Immunotherapy with this whole cell MIM-SIS adjuvanted autologous cancer vaccine has shown efficacy in rodent models of prostate cancer [26–28]. These data support the idea that MIM-SIS may be effective when used as an adjuvant for tissue vaccines in a clinical situation. With this approach, tumor cells are not cultured, avoiding the changes in antigenic profiles that occur *in vitro* as demonstrated by microarray analysis [29]. This autologous cancer vaccine also appears to be well-tolerated by cancer-bearing dogs [24].

With therapeutic cancer vaccines, the goal is to increase presentation of TAA to the immune system, thereby increasing activation of tumor-specific T cells, favoring the elimination and equilibrium stages of cancer immunoediting in the host [30]. The result is an enhanced ability of the immune system to destroy cancer cells, stop or slow the growth of cancer cells, or delay metastasis which can improve patient survival. The purpose of this study was to determine the *in vitro* effects of this whole cell adjuvanted autologous cancer vaccine on a canine monocyte cell line, and determine the effect on postoperative survival of dogs with metastatic HSA when compared to a historical control group of dogs with metastatic HSA treated with either surgery alone or surgery followed by standard anthracycline-based chemotherapy protocols.

## Results

### Mechanism of action assay

**Fig. 1:**
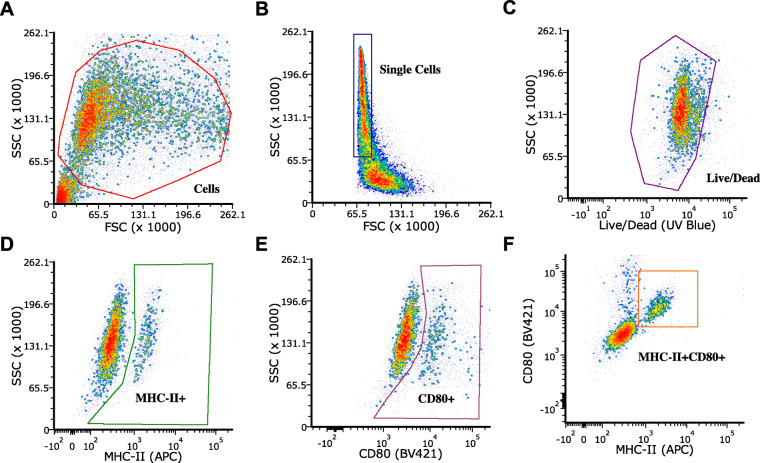
Flow Cytometry Gating Scheme. **a** Forward scatter/side scatter elimination of debris. **b** Single cell selection, gated on cells. **c** Viability stain (Live/Dead UV Blue) live cell selection, gated on single cells. **d** MHC-II+ (APC) single-stain cell selection, gated on living cells. **e** CD80+ (BV421) single-stain cell selection, gated on living cells. **f** MHC-II + CD80+ (APC, BV421) double-stained cell selection, gated on living cells

**Fig. 2:**
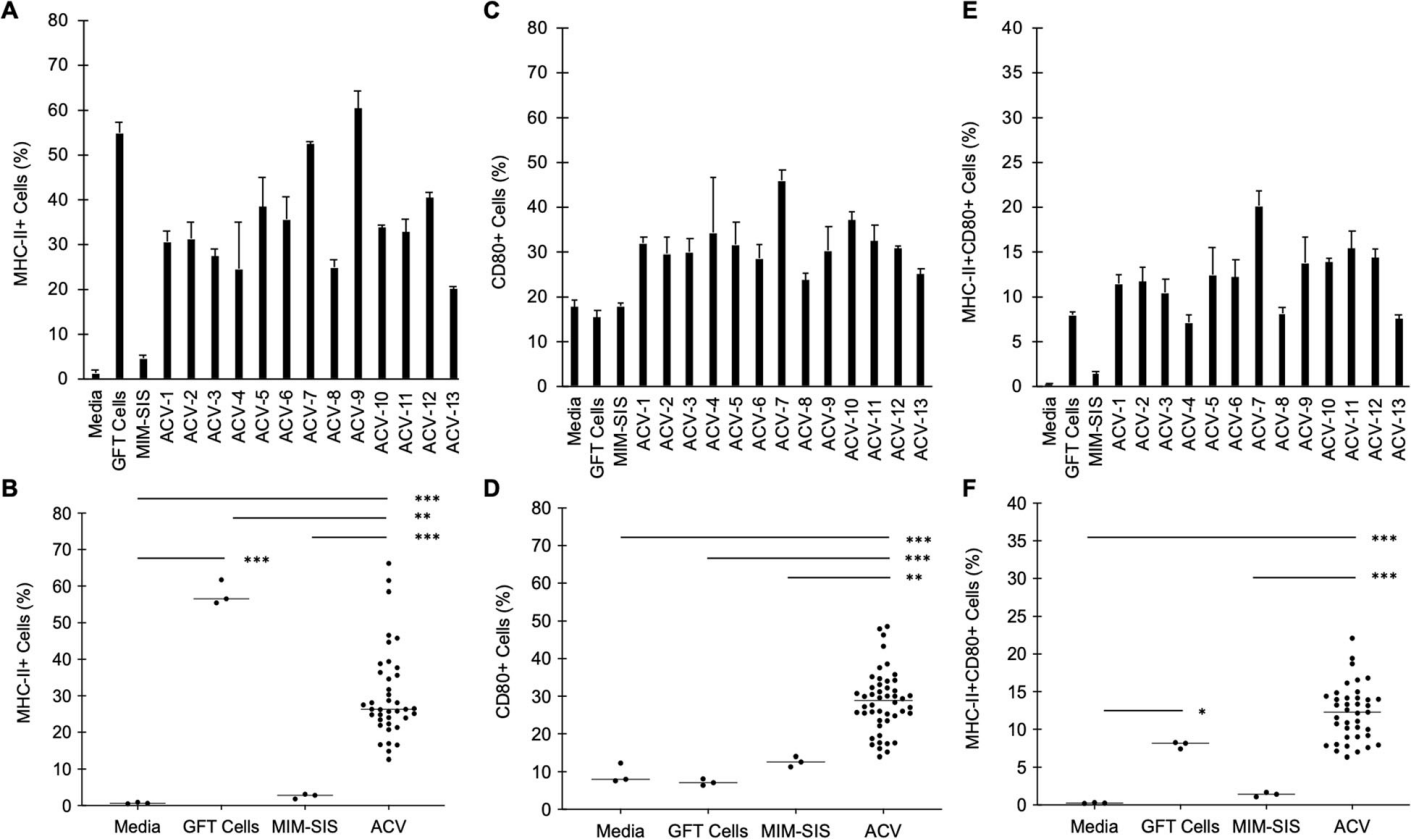
Antigen Presentation Assay, CD80 and MHC-II Expression. Thirteen autologous cancer vaccines (ACV), media only control, glutaraldehyde-fixed (GFT) cell only control and MIM-SIS only control. **a** MHC-II^+^ single stained cell percentage of total live cells, individual ACV preparations plotted; data represent mean ± SD, *n* = 3. **b** MHC-II^+^ single stained cell percentage of total live cells, pooled ACV preparations plotted; data represent mean ± SD, *n* = 3 (controls) and *n* = 39 (ACV). **c** CD80 + single stained cell percentage of total live cells, individual ACV preparations plotted; data represent mean ± SD, *n* = 3. **d** CD80^+^ single stained cell percentage of total live cells, pooled ACV preparations plotted; data represent mean ± SD, *n* = 3 (controls) and *n* = 39 (ACV). **e** MHC-II + CD80 + double stained cell percentage of total live cells, individual ACV preparations plotted; data represent mean ± SD, *n* = 3. (F) MHC-II + CD80 + double stained cell percentage of total live cells, pooled ACV preparations plotted; data represent mean ± SD, *n* = 3 (controls) and *n* = 39 (ACV). Statistical comparisons are based on one-way ANOVA, followed by post hoc Tukey’s pairwise comparisons. The asterisks denote statistical significance at the level of * *p* < 0.05, ** *p* < 0.01, *** *p* < 0.001. ANOVA, analysis of variance; SD, standard deviation

Surrogate autologous cancer vaccine preparations were formulated from 13 dogs with HSA of variable origin and stage (Table 1). These surrogate vaccines were utilized to assess their immunostimulatory potential *in vitro* in terms of their ability to stimulate antigen presentation (MHC-II) and co-stimulation (CD80) in canine DH82 cells evaluated via flow cytometry (Fig. 1). From this analysis, it was demonstrated that autologous cancer vaccines increased MHC-II surface expression ranging from 20.0-60.4% on single-stained cells (Fig. 2a-b), CD80 surface expression ranging from 23.7–45.9% on single-stained cells (Fig. 2c-d), and MHC-II/CD80 surface expression ranging from 7.2–20.1% on double-stained cells (Fig. 2e-f). Notably, autologous cancer vaccines (*n* = 13) were able to, on average, stimulate an up-regulation of MHC-II and CD80 by 48-fold as compared to media only (MHC-II + CD80 + cells: 12.19 ± 3.70% vs. 0.25 ± 0.06%; *p* < 0.001). As compared to autologous cells alone without MIM-SIS adjuvant, autologous cancer vaccines increased the expression of MHC-II and CD80 by 53.7% (MHC-II + CD80 + cells: 12.19 ± 3.70% vs. 7.93 ± 0.46%; *p* = 0.18). Lastly, as compared to MIM-SIS adjuvant only, autologous cancer vaccines increased the expression of MHC-II and CD80 by 9-fold (MHC-II + CD80 + cells: 12.45 ± 3.53% vs. 1.33 ± 0.36%; *p* < 0.001).

**Table 1:** Characteristics of dogs with hemangiosarcoma utilized for mechanism of action evaluation

Patient	Breed	Age (y)	Sex/Status^b^	Weight (kg)	Primary	Metastasis
16–034	Mixed	6	F/S	12.2	Subcutaneous	No
18–082	Golden retriever	NR^a^	M/C	40.9	Liver	Yes
19–039	Mixed	12	M/C	36.2	Subcutaneous	No
19–060	Boxer	7	M/I	29.5	Subcutaneous	No
19–195	Rottweiler	3	F/S	34.9	Bone^c^	No
19–199	Labrador retriever	12	M/C	NR	Spleen	No
19–261	Golden retriever	10	F/S	25.9	Spleen	No
20−008	Mixed	NR	M/C	12.2	Spleen	No
20−010	Coonhound	7	F/S	31.8	Spleen	No
20–020	Mixed	13	M/C	22.7	Subcutaneous	No
20–025	English Setter	14	M/C	31.6	Spleen	No
20–027	Mixed	10	M/C	24.0	Spleen	No
20–040	Mixed	10	M/C	41.5	Spleen	Yes

^a^*NR* Not reported

^b^*F *female, *M *male, *S *spayed, *C *castrated, *I *intact

^c^The submitting veterinarian did not pursue immunohistochemisty, so telangiectatic osteosarcoma is not excluded. However the pathologist did not find anything to support osteosarcoma in the tissue sectons examined

### Study population

#### Cases

There were 32 dogs with HSA identified within the Torigen database, and 8 dogs met the entry criteria (Table 2). Of the 24 dogs that were excluded, 10 had stage I or II disease, 9 dogs had incomplete medical records, and 5 dogs had no staging tests done by the attending veterinarian. Six different pure breeds were represented, including 2 (25%) German Shepherd Dogs and 1 (13%) each of golden retriever, Labrador retriever, great Dane, standard poodle and Australian cattle dog. There was 1 (13%) mixed breed dog. The mean age at diagnosis was 7.6 ± 1.71 years (range, 7–10 years). The mean patient weight was 39.5 ± 11.83 kg (range, 22.4–54.7 kg). There were 7 (88%) males (1 intact) and 1 (12%) spayed female. HSA was diagnosed within the spleen in 7 dogs (88%) and was subcutaneous in 1 dog (12%). Of the 7 dogs with splenic HSA, 4 (57%) were reported to have hemoperitoneum at the time of diagnosis. Disseminated disease included metastasis to the liver (*n* = 3), lungs (*n* = 3), right atrium (*n* = 1) and omentum (*n* = 1). The mean time from surgery until the starting the vaccine series was 17 ± 6 days.

#### Historical controls

There were 42 dogs in the surgery only group. Twelve different pure breeds were represented, including 9 (21%) German Shepherd Dogs 5 (12%) Labrador retrievers, 3 (7%) border collies, 2 (5%) each of boxers and beagles, and 1 (2%) each of American pit bull terrier, English setter, cane corso, golden retriever, Airedale terrier, Newfoundland, and rottweiler. There were 14 (33%) mixed breed dog. The mean age at diagnosis was 10.3 ± 2.00 years (range, 6–14 years). The mean patient weight was 30.2 ± 5.96 kg (range, 15.0–52.8 kg). There were 22 (52%) males (11 intact) and 20 (48%) females (6 intact). HSA was diagnosed within the spleen in all 42 dogs. The presence or absence of hemoperitoneum at the time of diagnosis was not reported in the data set provided. Disseminated disease included metastasis to the liver (*n* = 26), omentum (*n* = 8), lungs (*n* = 4), hepatic lymph nodes (n = 2), right atrium (*n* = 1) and pleura (*n* = 1).

There were 23 dogs in the maximum tolerated dose (MTD) chemotherapy group. group. Eight different pure breeds were represented, including 8 (35%) German Shepherd Dogs, 2 (9%) Labrador retrievers, and 1 (4%) each of golden retriever, cane corso, Brittany, beagle, dachshund, and Czechoslovakian wolf dog. There were 7 (30%) mixed breed dogs. The mean age at diagnosis was 10.0 ± 2.21 years (range, 7–14 years). The mean patient weight was 31.6 ± 9.84 kg (range, 5.1–47.0 kg). There were 14 (61%) males (12 intact) and 9 (39%) females (4 intact). HSA was diagnosed within the spleen in all 23 dogs. Information regarding hemoperitoneum at diagnosis was not described in the shared data set. Disseminated disease included metastasis to the liver (*n* = 16), omentum (*n* = 5) and lungs (*n* = 1). In the MTD chemotherapy group, standard anthracycline-based protocols were used, and 17 (74%) dogs were treated with doxorubicin, 3 (13%) dogs were treated with epirubicin, 2 (9%) dogs were treated with doxorubicin and cyclophosphamide, and 1 (4%) dog was treated with doxorubicin and dacarbazine.

The proportion of male dogs was significantly higher in the autologous cancer vaccine group when compared to the surgery only group (p < 0.05). The proportion of male dogs was not significantly different between the autologous cancer vaccine and MTD chemotherapy groups. There were no significant differences in variance of weights or ages among of the treatment groups.

### Clinical outcomes

#### Adverse events

In the 8 dogs treated with the adjuvanted autologous cancer vaccine, no episodes of anaphylaxis or other adverse events were reported following any of the 24 doses administered. In the dogs treated with MTD chemotherapy, 10 (43%) dogs were reported to have at least 1 adverse event, and 4 of the 10 dogs had multiple adverse events. Overall, 4 (17%) dogs required hospitalization for grade 3–4 bone marrow toxicity (*n* = 2) and grade 3 GI toxicity (*n* = 2).

#### Survival

Progressive HSA was the cause of death in all dogs that died during the follow-up period in both the historical control group and autologous cancer vaccine group. Four dogs were right-censored (1 dog treated with the autologous cancer vaccine and 3 dogs treated with surgery alone).

**Fig. 3:**
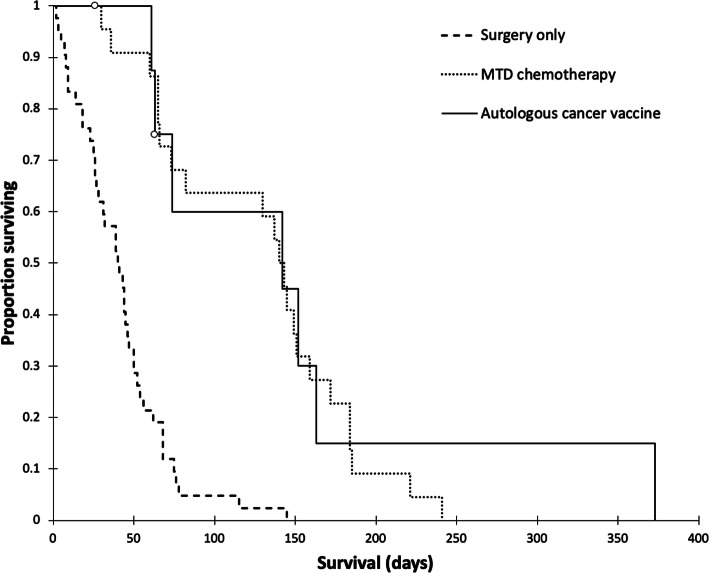
Kaplan-Meier survival curves for dogs with metastatic (stage III) hemangiosarcoma treated with surgery alone (*n* = 42), surgery followed by maximum tolerated dose chemotherapy (*n* = 23), and surgery followed by the autologous cancer vaccine (*n* = 8). Open circles represent right-censored observations

The median survival time for dogs treated with surgery alone was 41 days (range, 2 to 145 days). The median survival time for dogs treated with MTD chemotherapy was 142 days (range, 26 to 241 days). The overall median survival time for dogs treated with the autologous cancer vaccine was 142 days (range, 61 to 373 days). Dogs treated with the autologous cancer vaccine or MTD chemotherapy had significantly (*P < 0.001)* longer survival than dogs treated with surgery alone. There was no significant difference in survival between dogs treated with the autologous cancer vaccine and those treated with MTD chemotherapy (Fig. 3). The 1-year survival rate was 12.5% for dogs treated with the autologous cancer vaccine, and 0% for dogs treated with surgery alone or MTD chemotherapy. Summary data for the three patient groups are presented in Table  3.

**Table 2:** Characteristics of dogs with stage III hemagiosarcoma treated with surgery and the autologous cancer vaccine

Patient	Breed	Age (y)	Sex/Status^b^	Weight (kg)	Primary	Hemoabdomen
15–049	Great Dane	7	F/S	54.7	Spleen	Y
16−008	Standard poodle	6	M/C	22.4	Subcutaneous	
18–082	Golden retriever	NR^a^	M/C	40.9	Liver	NR
18–216	German shepherd dog	7	M/C	40.9	Liver	Y
19–023	German shepherd dog	6	M/C	54.5	Liver	N
19–120	Australian cattle dog	10	M/I	25.0	Spleen	Y
19–167	Labrador retriever	7	M/C	36.4	Spleen	Y
20–040	Mixed	10	M/C	41.5	Spleen	N

^a^*NR *Not reported

^b^*F* female, *M *male, *S *spayed, *C *castrated, *I *intact

**Table 3:** Summary data from 73 dogs with metastatic hemangiosarcoma

	Surgery alone *(n = 42)*	Surgery plus chemotherapy *(n = 23)*	Surgery plus autologous cancer vaccine *(n = 8)*
**Age, years (mean ± SD)**	10.3 ± 2.00	10.0 ± 2.21	7.6 ± 1.71
**Weight, kg (mean ± SD)**	30.2 ± 5.96	31.6 ± 9.84	39.5 ± 11.83
**Adverse events (%)**	0	43	0
**Hopitalizations (%)**	0	17	0
**Median survival time, days**	41	142	142
**Survival range, days**	2 to 145	26 to 241	61 to 373
**Alive at 1 year (%)**	0	0	12.5

## Discussion

Cancer is the leading cause of death in dogs [31]. Every year approximately 4 million dogs will be diagnosed with some form of cancer, and 50% of all dogs over the age of 10 will die as a result of developing the disease [31, 32]. A major driving force behind these statistics is that, unfortunately, the therapeutic approaches used for treating dogs with cancer has lagged behind that of humans, resulting in sub-optimal clinical outcomes [33]. Immunotherapy offers a new approach to cancer treatment by re-educating the patient’s immune system to combat the disease [34, 35]. This re-education process is accomplished by providing back stimulatory factors and tumor-associated antigens that allow for the host immune system to recognize the tumor as foreign. One such immunotherapeutic modality that is capable of performing such function is the autologous whole tissue cancer vaccine being developed by Torigen Pharmaceuticals [24].

Utilizing an *in vitro* assay of antigen presentation and DH82 canine monocyte-like cells, it was possible to highlight the mechanism of action of autologous cancer vaccines combined with MIM-SIS adjuvant. Major histocompatibility complex II (MHC-II) are molecules found on professional antigen-presenting cells (amongst others) that serve to present epitopes to T lymphocytes. Specifically, MHC-II molecules present longer peptide-based epitopes utilized for priming of CD4 + T helper cells. Notably, MHC-I molecules facilitate priming of CD8 + cytotoxic T-cells that would be more directly relevant in the context of cancer immunotherapy, but no commercially available or literature published canine specific antibody is available at present for diagnostics. Beyond MHC, CD80 (or CD86) is a co- stimulatory molecule that increases in expression in response to antigen and facilitates T-cell priming by co-ligation with CD28 T-cells. Together, MHC and CD80 represent two of the three required signals for T lymphocyte activation and differentiation; the third being a local cytokine environment. Accordingly, it is reasonable to rationalize that the ability to upregulate the expression of these surface molecules on canine monocytes could be indicative of greater potential T lymphocyte response *in vivo*. As such, this technique has been applied in numerous studies evaluating new vaccine technologies including recombinant antigens, novel adjuvants and variable formulation schemes [36–42]. Specifically, the extent of major histocompatibility complex II (MHC-II) and CD80 co-stimulatory molecules were up-regulated in response to incubation with vaccine or relevant controls. These signals are traditional markers of antigen-presentation and are correlated to the ability to stimulate T-cell activation *in vivo* [43–45]. Notably, the MHC-II signal is indicative of helper T-cell activation, not cytotoxic T-cell activation [46]; at this time, no validated canine-specific antibody is commercially available for MHC-I quantification, a marker of cytotoxic T-cell activation, via flow cytometry.

The results of the antigen presentation assay suggest that there is an immunostimulatory synergy between autologous cancer cells and MIM-SIS adjuvant. Specifically, while autologous cancer cells alone (GFT cells) are capable of stimulating a robust extent of antigen-presentation, they fail to provide the co-stimulatory signal that is absolutely required for T-cell activation. Moreover, MIM-SIS alone promotes only weak co-stimulatory responses via CD80 surface expression. It is only when autologous cancer cells and MIM-SIS adjuvant are combined that there is a 53.7% increase in dual signaling of MHC-II and CD80 on canine cells, and thus a more robust potential for immune activation. The results of this assay indicate that there is no clear correlation between antigen-presentation/co-stimulation and tumor stage evaluated in an *in vitro* setting. *In vivo*, there could absolutely be a difference based upon the immunological status of that individual patient, which is highly dependent on manner factors even beyond the tumor type and grade. The most dominant discrepancy between *in vitro* and *in vivo* evaluations of the immune response would be the immunosuppressive tumor environment (negative impact), as well as tumor mutational burden (positive impact). Ultimately, the ability to upregulate CD80 and MHC-II would be derived from a balance of these two factors. Accordingly, this evidence, combined with our previous pre-clinical evaluations [26–29], provides justification for further research and warrants initial clinical validation in canine models.

The population of dogs described herein was similar in age, weight, and male predominance described to previous reports of HSA [5, 11, 12, 14, 47]. The preponderance of German shepherd dogs observed herein has also been previously described [4, 11, 47, 48]. The dogs treated with immunotherapy in this study all tolerated the autologous cancer vaccine well, with no reported adverse events described. This group of dogs had an even lower proportion of adverse event rate than the 12% reported in a population of 93 dogs with various cancers treated with the autologous cancer vaccine immunotherapy after surgery [24]. This is in stark comparison to the observed proportion of adverse events (43%) and hospitalizations (17%) within the MTD chemotherapy group.

Although all of the deaths during the follow-up period were due to progressive HSA, the observed median survival time of 142 days for dogs with metastatic HSA represented a more than 3-fold increase over the 41 day median survival time in the historical control group treated with surgery alone, and is more than 2.5-fold longer than the recently reported 54 day median survival time following surgery alone [9]. The majority of dogs treated with the adjuvanted autologous cancer vaccine had hemoperitoneum at presentation. Although there were no available comparison data from the historical control group, recent findings that hemoperitoneum does not affect survival time of dogs with HSA [49], suggests hemoperitoneum would be unlikely to influence outcome in the dogs described herein.

There were several limitations of this study. First, due to lack of available specimens, the *in vitro* work was conducted with surrogate HSA tumor vaccines created from patients not included in the clinical case evaluations and utilized a canine monocyte-like cell line rather than monocytes isolated from each specific dog. Although the upregulation of MCH-II and CD80 was demonstrated in the canine model, the findings were not specific to the dogs studied for survival outcome. However, the advantage of using the DH82 cell line is standardization and replication to facilitate vaccine-to-vaccine comparisons. If patient-specific monocytes were used, there would be two variables within the assay.

Another limitation was the retrospective nature of case evaluations. Because tumor specimens for the autologous cancer vaccine creation were submitted from a variety of clinicians (oncologists, surgeons, and general practitioners), pre-operative cancer staging, and post-treatment follow-up varied between patients. This limited the evaluation of vaccine efficacy in this population of dogs to simple survival analysis. Evaluating time to progression is an objective measurement of clinical efficacy that is free from bias introduced by dog owners’ decisions on euthanasia. However, given the aggressive nature of HSA in dogs, the lengths of progression-free interval and overall survival are typically similar among different study populations [10, 15, 50, 51].

The small number of available dogs with stage III HSA treated with only surgery and the autologous cancer vaccine, and heterogeny among the cases, represent other limitations of the current study. Regardless, these preliminary findings are encouraging; the median survival time of vaccinated dogs was significantly longer than dogs treated with surgery alone. Results from a larger, prospective study will be needed to confirm these preliminary findings. In addition to treating dogs with metastatic HSA, studies of dogs with stage I and stage II are also warranted, as the adjuvanted autologous cancer vaccine may be more effective in dogs with a lower cancer burden, and vaccine effects on preventing metastatic disease could be demonstrated.

Compared with other techniques for creating autologous tumor vaccines, the method used herein to create the adjuvanted autologous cancer vaccine does not require culturing tumor cells, which allows for rapid manufacturing of the patient-specific vaccine. Culturing tumor cells increases the chances for microbial contamination of the vaccine product. Using mechanical dissociation of tumor tissue also preserves TAA which may induce a more robust immunologic response.

The autologous cancer vaccine protocol used here, administering 3 weekly subcutaneous autologous cancer vaccine doses, allows a patient with stage III HSA to complete the course of therapy quickly, compared to dogs undergoing a standard anthracycline (doxorubicin or epirubicin) chemotherapy protocol receiving an intravenous injection every 3 weeks for 5 to 6 doses [11]. The autologous cancer vaccine also provides a significant survival advantage over surgery alone for dogs with stage III HSA, with minimal risk for adverse events. Many dog owners are concerned about adverse events associated with chemotherapy [20], and the anthracycline drugs have been associated with acute allergic reactions, GI upset, neutropenia, thrombocytopenia, and rarely cardiotoxicity [10, 12, 19, 52–60]. In the present study, adverse events were common in the historical control group treated with standard anthracycline chemotherapy. Additionally, for many dog owners, the time and financial commitments associated with chemotherapy for stage III HSA are not feasible, and the outcome expectations also factor into the decision-making process [61]. Treatment with the autologous cancer vaccine as reported here, and previously described, has a low risk of adverse events [24], and may be more affordable than chemotherapy.

This method of generating an adjuvanted autologous cancer vaccine represents an individualized form of immunotherapy, presenting a range of tumor-specific and host-specific antigens to the patient’s immune system, that may be appropriate for any solid tumor where sufficient cells can be collected for vaccine preparation. This is in contrast to other immunotherapy options, such as the canine melanoma vaccine and canine osteosarcoma vaccine, which present a single antigen to the patient’s immune system (human tyrosinase and HER2/neu, respectively) [62, 63].

## Conclusions

The data presented herein demonstrate that immunotherapy with the adjuvanted autologous cancer vaccine is capable of up-regulating MHC-II and CD80 in cultured canine monocyte-derived cells, which are important stimulatory molecules in generating an immune response, and the vaccine improves survival time in dogs with metastatic (stage III) HSA when compared to surgical treatment alone. The autologous cancer vaccine-treated dogs had survival similar to those dogs treated with maximum tolerated dose chemotherapy without any observed adverse events. This autologous cancer vaccine represents an effective form of individualized immunotherapy that is an appealing option for dog owners not wanting to pursue adjuvant chemotherapy for HSA.

## Methods

### Materials

All reagents were used as obtained from commercial sources without further purification. DH82 canine malignant histiocytoma cells (macrophage-like, CRL-10,389) were purchased from ATCC (Manassas, VA, USA). Anti-CD80 (Brilliant Violet 421, clone 16-10A1) was obtained from BioLegend (San Diego, CA, USA). Anti-MHC-II (APC, YKIX334.2) and Live/Dead Fixable Blue Stain Kit (L34962) were obtained from Thermo Fisher Scientific (Waltham, MA, USA).

### DH82 antigen presentation assay

The ability of autologous cancer vaccines to facilitate antigen presentation (MHC-II) and co-stimulation (CD80) was evaluated in-vitro using DH82 canine macrophages and flow cytometry. DH82 cells were maintained at 37 °C, 5% CO_2_/95% air atmosphere and approximately 85% relative humidity. DH82 cell culture media consisted of Dulbecco’s Modified Eagles Media, 15% heat-inactivated fetal bovine serum (VWR, Radnor, PA, USA), 1% SG-200 (GE Healthcare, Chicago, IL, USA), 1% sodium pyruvate (GE Healthcare), 1% antibiotic-antimycotic (Thermo Fisher Scientific). Prior to performing the assay, DH82 cells were seeded overnight at 2.5e5 cells/well in 24-well non-treated cell culture dishes. The antigen presentation assay was performed by incubating autologous cancer vaccine preparations (0.25e6 cells/well, 0.5 mg/mL MIM-SIS) or relevant controls with cells for 48 hours (performed in triplicate). Autologous cancer vaccines were prepared for thirteen unique HSA samples (Table 1) as previously described [24], from submitted patient samples with a sufficient amount of tissue available after creation of their vaccine for clinical use. After 48 hours, cells are harvested and prepared for analysis by flow cytometry using the following stains: Live/Dead Fixable, anti-CD80 Brilliant Violet 421 and anti-MHC-II APC. Cells were analyzed using a Becton-Dickinson LSR II flow cytometer (Franklin Lakes, NJ, USA) at the University of Connecticut Health Center Flow Cytometry Core (Farmington, CT, USA) with the FCS Express 7 software package (DeNovo Software, Pasadena, CA, USA). Fluorescence minus one (FMO) controls were utilized to established gating scheme.

### Case selection

The case accession database at Torigen Pharmaceuticals, Inc. was queried to identify dogs diagnosed with stage III HSA between January 2015 and January 2020 that were treated with surgery followed by the adjuvanted autologous cancer vaccine only.

Dogs were eligible for inclusion in this study if they had a histopathological diagnosis of non-cutaneous HSA and evidence of distant metastasis (i.e., stage III disease) identified via imaging, cytopathology or histopathology, and were treated by surgery and the adjuvanted autologous cancer vaccine only. Dogs were excluded from study if they were diagnosed with cutaneous HSA, had visceral, subcutaneous or intramuscular HSA with no evidence of metastasis (i.e., stage I or II), did not receive all three doses of the autologous cancer vaccine, received adjuvant chemotherapy, or had incomplete outcome information available. Histopathologic diagnosis was reported by board-certified veterinary pathologists via commercial laboratory services. Patient data collected included signalment, body weight, histology results, primary tumor location, adverse events reported after autologous tumor vaccine administration, and survival from the time of tumor removal. Follow up information on each dog was obtained through direct communication with the submitting veterinarian. Adverse events were classified based on the Veterinary Cooperative Oncology Group common terminology criteria for adverse events (VCOG-CTCAE) [64].

### Historical controls

The historical controls cases were dogs identified between 2011 and 2018 with stage III HSA that were either treated with surgery alone, or surgery followed with standard maximum tolerated dose (MTD) anthracycline-based chemotherapy. The outcomes of these dogs have been previously published [10], and the authors provided a raw data set which included signalment, adverse events and hospitalization reported after chemotherapy administration, and survival from the time of tumor removal. Adverse events were classified based on the VCOG-CTCAE [64].

### Vaccine protocol

Preparation of the adjuvanted autologous cancer vaccine has been described elsewhere [24]. Briefly, unfixed tumor tissue was mechanically dissociated, fixed with glutaraldehyde, and following multiple washing steps, the fixed cells were combined with MIM-SIS to create the final vaccine product. Veterinarians were instructed to give the vaccine as a subcutaneous injection, as three 1 mL doses, at weekly intervals. The attending veterinarian was advised to monitor the dog for acute AE for 30 minutes after each of the three injections. At hospital discharge, dog owners were informed of possible vaccine reactions, and instructed to report any observed abnormalities immediately upon their occurrence. Written owner informed consent was obtained before the vaccine was administered.

### Statistical analysis

In-vitro data are expressed as mean ± standard deviation (SD). Means of multiple groups were compared with the one-way analysis of variance (ANOVA), followed by post hoc Tukey’s pairwise comparisons. All probability values are two-sided, and values of *p* < 0.05 were considered statistically significant. Statistical analyses were carried out using the GraphPad Prism 7 software package.

Among treatment groups, the variance of continuous variables was compared using Levene’s test, and proportions were compared using the Marascuilo procedure. Dogs were right-censored from survival analysis if they were lost to follow-up, died from an unrelated cause, or were still alive at the time of data analysis. Progression-free survival was not calculated due to insufficient information in the database or attending veterinarian medical records. Survival estimates were generated using the Kaplan-Meier method, and survival curves were compared using the Log-Rank test. Statistical testing was done using XLSTAT Life Science (Addinsoft, 2020, New York, USA). *P* values < 0.05 were used to indicate statistical significance. Results are reported as mean ± standard deviation unless otherwise noted.

## Data Availability

The datasets generated and/or analyzed during the current study are available from the corresponding author on reasonable request.

## Abbreviations

ABC: ATP-binding cassette
GI: Gastrointestinal
HSA: Hemangiosarcoma
IP: Intraperitoneal
MIM-SIS: Matrix immunomodulator, small intestine submucosa-derived
MTD: Maximum tolerated dose
TAA: Tumor-associated antigens
